# Global seroprevalence of *Toxoplasma gondii* infection among patients with mental and neurological disorders: A systematic review and meta‐analysis

**DOI:** 10.1002/hsr2.1319

**Published:** 2023-06-05

**Authors:** Habtye Bisetegn, Habtu Debash, Hussen Ebrahim, Naunian Mahmood, Alemu Gedefie, Mihret Tilahun, Ermiyas Alemayehu, Ousman Mohammed, Daniel Getacher Feleke

**Affiliations:** ^1^ Department of Medical Laboratory Sciences, College of Medicine and Health Sciences Wollo University Dessie Ethiopia; ^2^ Department of Zoology University of Sargodha Sargodha Pakistan; ^3^ Department of Microbiology, Immunology and Parasitology, College of Health Sciences Addis Ababa University Addis Ababa Ethiopia

**Keywords:** global prevalence, neuropsychiatric disorder, psychiatric patients, *Toxoplasma gondii*, toxoplasmosis

## Abstract

**Background and Aim:**

Toxoplasmosis is the most widespread zoonotic disease that affects one‐third of the world's population, and imposes a major public health problem worldwide. This study aimed to assess the prevalence of toxoplasmosis among patients with neuropsychiatric patients.

**Methods:**

Electronic databases PubMed, Google Scholar, Web of Science, Research Gate, and Scopus were thoroughly searched from February to March 2022 to identify all relevant studies. The quality of studies was evaluated using the Newcastle−Ottawa quality scale for case‐control and cross‐sectional studies. Statistical analysis was done using STATA version 12 software. A random effect model was used to compute the global pooled seroprevalence of *Toxoplasma gondii* infection. Heterogeneity was quantified by using *I* ^2^ value. Subgroup analysis was done, and publication bias was assessed using a funnel plot and Egger's test.

**Result:**

Of 1250 studies, 49 containing 21,093 participants and conducted in 18 countries were included. The global pooled seroprevalence of *T. gondii* IgG antibody was 38.27% (95% CI: 32.04−44.9) among neuropsychiatric patients and 25.31% (95% CI: 21.53−29.08) in healthy controls with substantial heterogeneity of 98.3%. The prevalence of *T. gondii* IgG antibody was higher in males (17.52%) than in females (12.35%) neuropsychiatric patients. The highest pooled prevalence of *T. gondii* IgG antibody was in Europe (57%) followed by Africa (45.25%) and Asia (43%). Time based analysis showed the highest pooled prevalence of *T. gondii* IgG antibody in 2012−2016 (41.16%).

The global pooled seroprevalence *T. gondii* IgM antibody among neuropsychiatric patients and healthy controls was 6.78% (95% CI: 4.87−8.69) and 3.13% (95% CI: 2.02−4.24), respectively.

**Conclusion:**

The pooled prevalence of chronic and acute *T. gondii* infection among neuropsychiatric patients was 38.27% and 6.78%, respectively. This showed a high burden of toxoplasmosis among neurological and psychiatric patients and urges routine screening of those patients and providing appropriate treatment. It also indicates the need for different stakeholders to develop targeted prevention and control strategies for *T. gondii* infection.

## 1 INTRODUCTION

Toxoplasmosis is the most widespread zoonotic disease caused by *Toxoplasma gondii*, which is a single‐cell obligate intracellular apicomplexan protozoan parasite that can invade and replicate inside all nucleated cell types of warm‐blooded animals. *T. gondii* infects a one‐third of the world's population with a seroprevalence of ranging from 10% to more than 90%.^1^, ^2^ It causes potentially serious disease in human and animals resulting in a major public health and economic burden in the world. *T. gondii* is reported to have a wide spectrum of intermediate hosts, which includes human, sheep, pig, rodents, and birds and harbors the asexual tachyzoite which is the active and lytic form of the parasite that cause life‐threatening diseases. The tissue cyst forms of the parasite is a slow‐growing stage capable of building cysts mostly in the brain and muscle tissues.^3^, ^4^, ^5^ Human beings acquire *T. gondii* infection through ingestion of the tissue cysts in raw or undercooked meat containing the latent cyst, sporulated oocysts in contaminated water or food, and congenitally from mother to child.^6^, ^7^ High prevalence of toxoplasmosis is reported in Africa, Southeast Asia, Middle East, Central/Eastern Europe, and Latin America. Variable prevalence of *T. gondii* infection was reported in Asia (13.3%−85.3%), Europe (40%−76%), Africa (21.74%−74.8%), North America (7.3%−26.5%). In immunocompetent individuals, *T. gondii* infection is usually asymptomatic and majority of the parasite is cleared during acute phase infection. Surviving parasites persist as slow‐growing bradyzoite tissue cysts, most abundant in tissues with limited immune surveillance, including brain, eye, cardiac, and skeletal muscle.^3^ In symptomatic cases, *T. gondii* is associated with lymphadenopathy, non‐flue like symptoms, toxoplasmic retinochoroiditis, ocular toxoplasmosis, and toxoplasmic encephalitis.^7^ It also causes sever opportunistic infection in pregnant and other immunocompromised patients due to reactivation of infection in the central nervous system (CNS).^8^ The synergetic effect of parasite growth, tissue damage, inflammatory response, host and parasite genotype results in the severity of the diseases.^4^

*T. gondii* is able to produce long‐lasting infection and persists in the CNS invading neurons, and functional glial cells leading to various neurological and mental disorders.^9^ *T. gondii* pass the impermeable blood brain barrier (BBB) through several mechanisms such as monocyte and other infected myeloid derived cells extravasate from capillaries to the brain, trans‐endothelial migration through attachment of the parasite to CD11b/ICAM1integrins, paracellular entry of the parasite into the CNS through actin‐myosin motors, and endothelial lysis.^9^, ^10^

Several neurological and mental disorders have been reported in patients with toxoplasmosis such as Alzheimer's disease (AD), schizophrenia, bipolar disorders, generalized anxiety disorder (GAD), obsessive‐compulsive disorder, suicidality, Parkinson's disease, epilepsy, depression, dysphoria, and sexual promiscuity.^11^, ^12^, ^13^, ^14^, ^15^, ^16^

There are several postulates regarding the chronic neuropathologic mechanisms in toxoplasmosis. These could be downregulation of the glutamate receptor (GLT‐1) expression leading to increased level of extracellular glutamate and excitatory glutamatergic signaling and neural damage, and alteration of glutamate decarboxylase 67 (GAD67) which consequently leads to decreased GABAergic synaptic activities. In addition, parasite induced inflammation and overexpression of several cytokines by the infection can contribute to the onset of seizure in *T. gondii* infected individuals.^4^ *T. gondii* infection may also lead to impaired catecholamine metabolism resulting psychological, behavioral, and motor changes in infected individuals.^17^

Several studies reported that there is a significant association between *T. gondii* and the various psychiatric and neurological disorders. A recent systematic review reported schizophrenia to be the most frequently linked psychiatric disorder with *T. gondii* infection.^18^ A meta‐analysis conducted on the effect of *T. gondii* on epilepsy reported *T. gondii* to the main risk factor for epilepsy.^19^ Another systematic review and meta‐analysis that evaluated the association between *T. gondii* infection and Parkinson and Alzheimer diseases found a positive association between the parasite and the neuropsychiatric diseases.^20^

Although several studies have reported the seroprevalence of *T. gondii* among neuropsychiatric patients in different parts of the world, studies reporting the pooled prevalence of *T. gondii* at the global level are lacking. Therefore, this systematic review and meta‐analysis was aimed to systematically review and determine the global burden and impact of *T. gondii* infection among patients with neuropsychiatric disorders and the importance of screening patients with neuropsychiatric disorders for toxoplasmosis to improve the quality of life of the patients.

## 2 MATERIALS AND METHODS

### 2.1 Search strategy and study selection

All articles regarding *T. gondii* infection were retrieved through systematic search of electronic databases such as PubMed/Central, Google Scholar, Web of Science, Research Gate, and Scopus from February to March 2022. The keywords used in this study includes; (1) Prevalence, seroprevalence, and magnitude, (2) Toxoplasmosis, *T. gondii*, (3) psychiatric disorder, mental disorder, neurologic disorder, (4) world, Africa, Asia, Europe, Middle East, America, Russia. These keywords were also used in combination for retrieving the studies. Cited sources from these papers were used as a resource for finding other related studies. Duplicates were removed and three independent reviewers (H. B., D. G. F., H. D.) continued to screen the title and abstract of all potentially eligible studies. Then the full text of potentially eligible studies that reported the prevalence of *T. gondii* infection among patients with neuropsychiatric disorders were added to the collections for extraction. Disagreements among authors during data extraction were resolved by discussion.

### 2.2 Eligibility criteria

Original articles that reported the seroprevalence of *T. gondii* infection among patients with any neuropsychiatric disorders were included. Studies reported only in English were included. However, non‐English articles which had abstracts in English that contained the required data for extraction were also included. On the other hand, studies reported the seroprevalence of *T. gondii* infection among non‐neuropsychiatric human study participants and nonhuman subjects (animals, rodents) were excluded. Furthermore, review articles, case reports, and letters to the editor were also excluded.

### 2.3 Outcome variables

The outcome variable for this study is the global pooled seroprevalence of *T. gondii* infection (*T. gondii* IgG and IgM antibodies) among neuropsychiatric patients.

### 2.4 Data extraction and quality assessment

Data from the eligible studies were extracted by three reviewers (H. B., N. M., and H. E.) independently in a Microsoft Excel sheet. The information extracted from each study includes name of the first author, publication year, country, continent, study design, sample size, number of male and female participants, diagnostic methods, form of neuro‐psychiatric disorder, prevalence of IgG, prevalence of IgM among case and control group. Quality of the included studies was assessed using the Newcastle−Ottawa quality scale for a case‐control and cross‐sectional studies.^21^, ^22^

### 2.5 Statistical analysis

The data extraction was done using Microsoft Excel worksheet and the meta‐analysis was done by using STATA version 12 software with the metan commands. The outcome of this study was reported as percentage prevalence and exact 95% confidence interval (CI). The point estimate and 95% CI of seroprevalence of *T. gondii* infection for all the included studies were calculated. Due to the high heterogeneity reported, the global pooled seroprevalence of *T. gondii* infection among neuropsychiatric patients and healthy controls was calculated using a random effect model.^23^ The Cochrane's *Q* test and *I* ^2^ statistics provide an estimate of the percentage of variability in effect estimates that is due to heterogeneity rather than chance alone were used to assess the heterogeneity. The *I* ^2^ statistics (percentage of total variability due to heterogeneity) indicates the heterogeneity and its value of 25%, 50%, and 75% corresponds to low, moderate, and high heterogeneity, respectively.^24^ Subgroup analysis for the primary outcome was performed in sex, region, country, and year of publication. Moreover, publication bias was assessed by visual observation of the symmetry of the funnel plot, and Egger's test statistics.^25^ Sensitivity analysis was done to assess the impact of a single study on the overall pooled effect size.

## 3 RESULT

### 3.1 Selection and identification of studies

**Figure 1:**
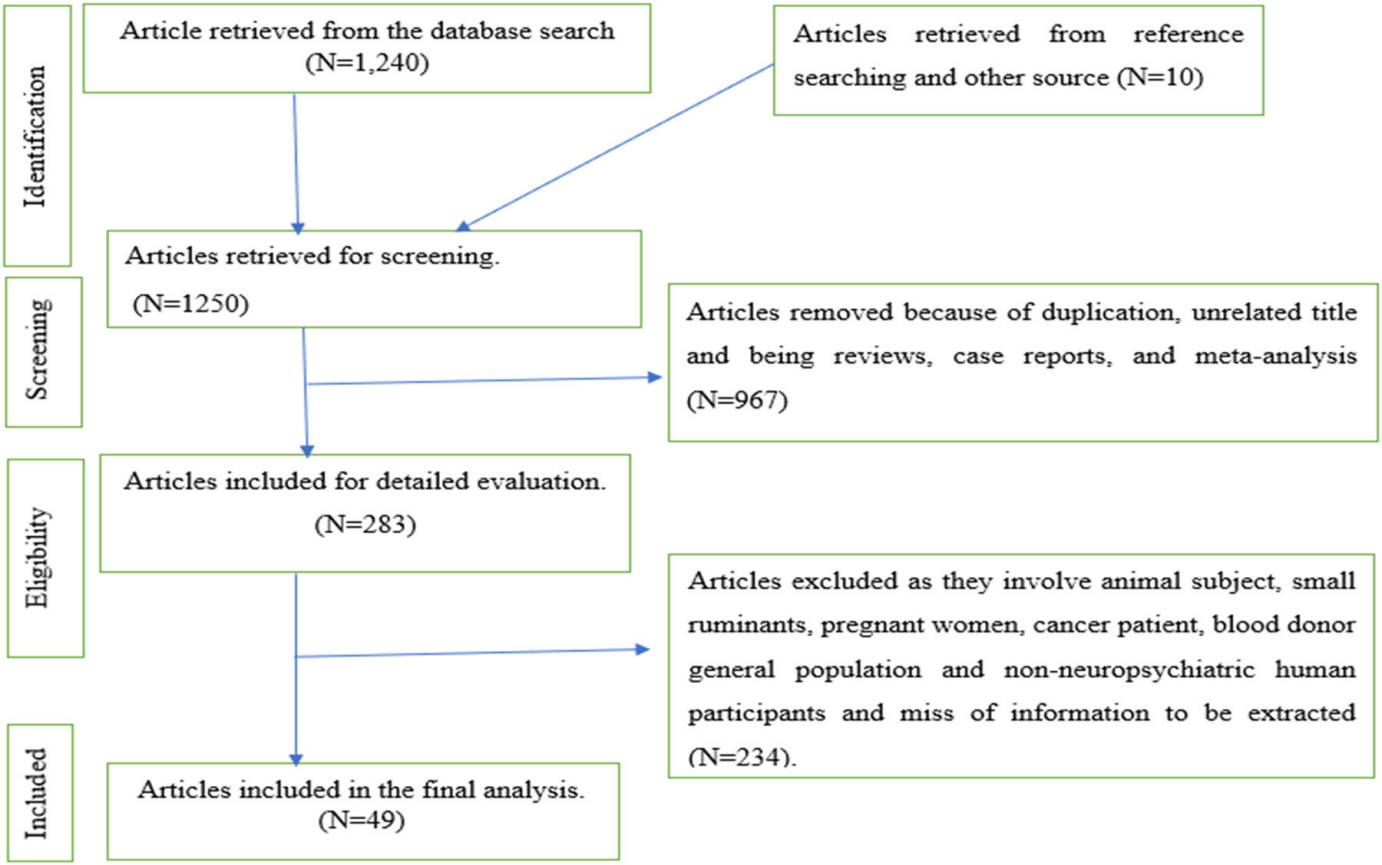
Flow diagram for the selection of eligible studies.

A total of 1250 articles were retrieved by systemic search and other methods. Of the total retrieved studies, 967 studies were removed due to duplication, reviews, case reports, letters to the editor, and meta‐analysis. Then the abstract and full text of 283 articles were evaluated in detail based on the eligibility criteria and 234 articles were removed due to failure to fulfill the inclusion criteria. Finally, 49 eligible studies were included in this systematic review and meta‐analysis. The preferred reporting items for Systematic Review and Meta‐analysis (PRISMA checklist 2009) was followed (Figure 1).^26^

### 3.2 Study characteristics

In this systematic review and meta‐analysis, 49 studies conducted in 18 countries from the four continents (Asia, North America, Africa, and Europe) that consisted of 8592 neuropsychiatric patients and 12,501 healthy controls were included. The included studies were conducted between 2006 and 2022. The majority of the studies were from Iran (12 studies) and Mexico (10 studies). The number of studies was higher in Asia followed by North America and Africa. In terms of epidemiological design, majority of the studies (44/49) were case‐control. All the included studies used ELISA as their diagnostic method. Neurological and mental disorders such as autism, schizophrenia, suicide victims, suicidal ideation, depression, Parkinson's disease, anxiety, bipolar disorders, psychiatric, and neurodevelopmental disorders were reported to be associated with *T. gondii* infection in the included studies (Table 1).

**Table 1:** Characteristics of the included studies.

Author/year/reference	Country	Study design	Neuropsychiatric disorders	Cases					Controls				
				Sample size	IgG case	IgG (%)	IgM case	IgM (%)	Sample size	IgG case	IgG (%)	IgM case	IgM (%)
Alvarado‐Esquivel (2017)^27^	Mexico	Case control	Neurological disorder	344	25	7.3	5	14.3	344	35	10.2	NA	NA
Alvarado‐Esquivel (2016)^28^	Mexico	Case control	Depression	89	11	12.4	NA	NA	356	22	6.2	4	1.1
Daryani (2010)^29^	Iran	Case control	Schizophrenia	80	28	35	9	11.2	99	25	25.3	11	11.1
Chen (2019)^30^	China	Case control	Psychiatric disorders	798	106	13.3	33	4.1	681	64	9.4	13	1.9
Sapmaz (2019)^31^	Turkey	Case control	Depression	37	8	21.6	NA	NA	36	2	5.6	NA	NA
Akgul (2021)^32^	Turkey	Case control	Schizophrenia	117	63	53.8	NA	NA	120	33	27.5	NA	NA
Alvarado‐Esquivel (2006)^33^	Mexico	Case control	Psychiatric disorders	137	25	18.2	NA	NA	180	16	8.9	NA	NA
Huseein (2020)^34^	Egypt	Case control	Schizophrenia and bipolar disorder	110	57	51.8	NA	NA	50	15	30	NA	NA
Muflikhah (2018)^35^	Indonesia	Case control	Schizophrenia	94	65	69.1	NA	NA	64	42	65.6	NA	NA
Akaltun (2018)^36^	Turkey	Case control	OCD and GAD	120	40	33.3	NA	NA	60	6	10	NA	NA
Elsaid (2014)^37^	Libya	Case control	Psychiatric disorders	300	151	50.3	NA	NA	300	99	33	NA	NA
Alvarado‐Esquivel (2011)^38^	Mexico	Case control	Schizophrenia	50	10	20	2	4	150	8	5.3	NA	NA
Alvarado‐Esquivel (2013)^39^	Mexico	Case control	Psychiatric disorders	156	7	4.5	3	1.9	127	10	7.9	3	2.4
Rashno (2016)^40^	Iran	Case control	Alzheimer's disease	87	58	66.5	NA	NA	87	49	56.3	NA	NA
Kheirandish (2016)^41^	Iran	Case control	Schizophrenia and bipolar disorder	170	103	63.5	14	8.2	170	65	38.2	8	4.7
Bakre (2015)^42^	Iraq	Case control	Schizophrenia	93	30	32.3	9	9.7	93	4	4.3	1	1.1
Dogruman (2009)^43^	Turkey	Case control	Schizophrenia	88	42	47.7	NA	NA	88	19	21.6	NA	NA
Mouhawess (2020)^44^	Lebanon	Case control	Schizophrenia	150	56	37.5	NA	NA	150	1	0.7	NA	NA
Esshili (2016)^45^	Tunisia	Case control	Schizophrenia	246	184	74.8	NA	NA	117	63	53.8	NA	NA
Fallahi (2017)^46^	Iran	Case control	Parkinson's disease	115	61	53	NA	NA	115	64	55.6	NA	NA
Stepanova (2019)^47^	Russia	Case control	NA	115	62	40	NA	NA	152	39	25	NA	NA
Alvarado‐Esquivel (2019)^48^	Mexico	Case control	Bipolar disorder	66	6	9.1	NA	NA	396	22	5.6	4	1
Hamdani (2013)^49^	France	Case control	Bipolar disorder	110	85	76.9	NA	NA	106	51	48.2	NA	NA
Hamidinejat (2010)^50^	Iran	Case control	Schizophrenia	134	71	53	6	4.5	48	14	29.2	2	4.2
Alipour (2011)^51^	Iran	Case control	Schizophrenia	62	42	67.7	NA	NA	62	23	37.1	NA	NA
Abdollahian (2017)^52^	Iran	Case control	Schizophrenia	350	164	46.9	17	4.85	350	120	34.3	3	0.9
James (2013)^53^	Nigeria	Case control	Psychiatric disorders	140	43	30.7	10	7.14	140	25	17.9	12	8.6
Abdelaal (2016)^54^	Egypt	Case control	Neuropsychiatric disorder	230	50	21.7	NA	NA	60	7	11.7	NA	NA
Zaki (2016)^55^	Saudi Arabia	Case control	Neuropsychiatric disorder	162	58	35.8	10	6	162	24	14.8	6	3.7
Khademvatan (2014)^56^	Iran	Case control	Schizophrenia	100	34	34	NA	NA	200	53	26.5	NA	NA
Alvarado‐Esquivel (2016)^57^	Mexico	Case control	Anxiety and depressive disorder	65	15	23.1	4	6.2	260	18	6.9	10	3.8
Kezai (2020)^58^	Algeria	Case control	Schizophrenia	70	49	70	NA	NA	70	37	52.9	NA	NA
Oskouei (2014)^59^	Iran	Case control	Parkinson's disease	75	64	85.3	NA	NA	75	68	90.3	NA	NA
Markovitz (2015)^60^	United state	Cross‐sectional	Anxiety disorders	484	128	26.5	NA	NA	NA	NA	NA	NA	NA
Grada (2022)^61^	Romania	Case control	Psychiatric disorders	308	209	67.9	NA	NA	296	160	54.1	NA	NA
Nasirpour (2020)^62^	Iran	Case control	Depression	87	52	59.8	NA	NA	87	49	56.3	NA	NA
Alvarado‐Esquivel (2021)^63^	Mexico	Case control	Suicidal ideation	306	37	12.1	10	3.3	1739	134	7.7	NA	NA
Alvarado‐Esquivel (2021)^64^	Mexico	Case control	Suicidal ideation	224	34	15.2	5	2.23	1199	118	9.8	NA	NA
Alvarado‐Esquivel (2021)^65^	Mexico	Cross‐sectional	Suicide victims	87	7	8	NA	NA	NA	NA	NA	NA	NA
Achaw (2019)^66^	Ethiopia	Case control	Psychiatric disorders	152	51	33.6	1	1.3	152	25	16.4	6	3.9
Cong (2015)^67^	China	Case control	Psychiatric disorders	445	77	17.3	14	3.2	445	55	12.4	10	2.3
Oana (2019)^68^	Romania	Case control	Schizophrenia and psychotic disorder	91	40	44	NA	NA	206	73	35.4	NA	NA
Olariu (2017)^69^	Romania	Cross‐sectional	Psychiatric disorders	214	117	54.7	NA	NA	NA	NA	NA	NA	NA
Shehata (2016)^70^	Egypt	Cross‐sectional	Neurodevelopmental disorder	188	94	50	31	16.5	NA	NA	NA	NA	NA
Ebadi (2014)^71^	Iran	Case control	Schizophrenia	152	81	53.2	48	31.5	152	63	41.4	30	19.7
Ansari‐Lari (2017)^72^	Iran	Case control	Schizophrenia	99	42	42.4	NA	NA	152	41	27	NA	NA
Xiao (2010)^73^	China	Case control	Psychiatric disorders	547	62	11.3	NA	NA	2634	329	12.5	NA	NA
Prandota (2015)^74^	Egypt	Cross‐sectional	Autism	46	11	23.9	NA	NA	NA	NA	NA	NA	NA
Esnafoglu (2017)^75^	Turkey	Case control	Autism	102	3	2.9	NA	NA	51	1	2	NA	NA

Abbreviations: GAD, generalized anxiety disorder; NA, not available; OCD, obsessive‐compulsive disorder.

### 3.3 Seroprevalence of chronic *T. gondii* infection (IgG antibody) among neuropsychiatric patients and healthy controls

**Figure 2:**
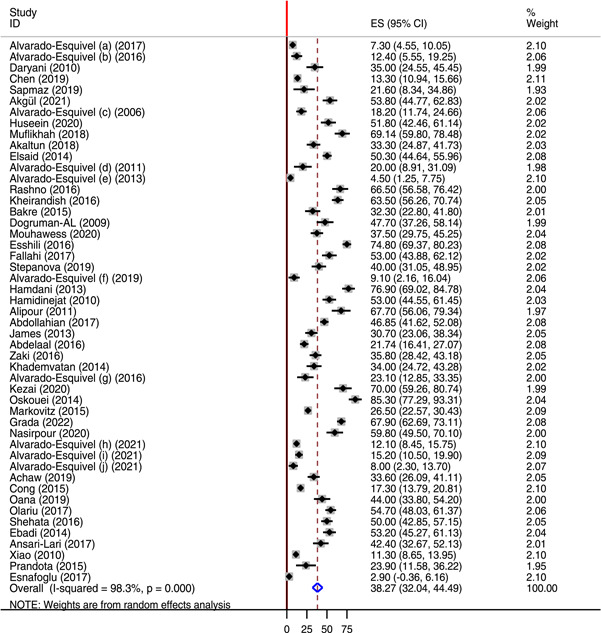
Forest plot showing the global pooled seroprevalence of *Toxoplasma gondii* IgG antibody among neuropsychiatric patients from 2006 to 2022.

**Figure 3:**
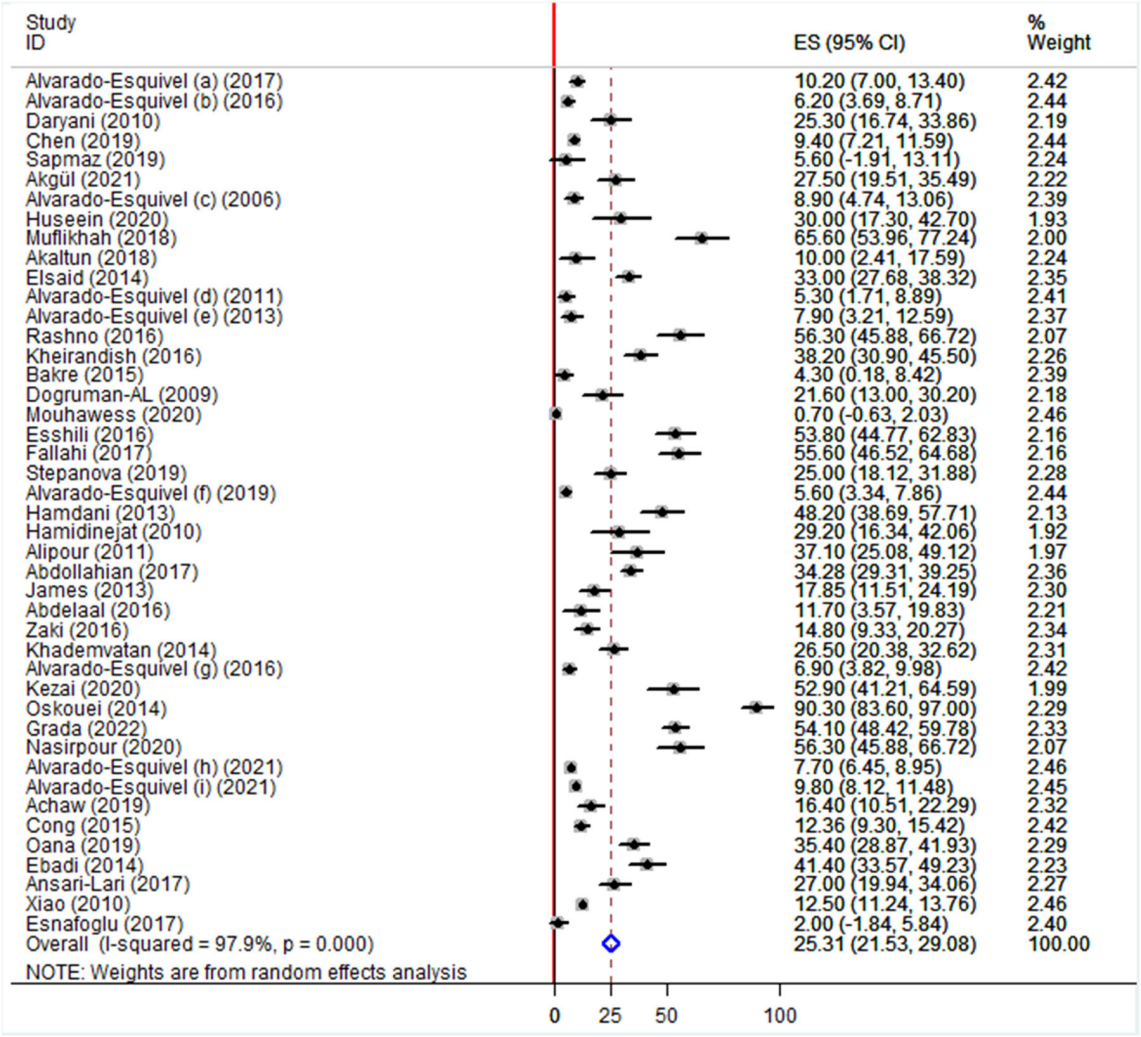
Forest plot showing the global seroprevalence of *Toxoplasma gondii* IgG antibody among apparently healthy control from 2006 to 2022.

Overall, the seroprevalence of *T. gondii* IgG among patients with neuropsychiatric disorders was variable, ranged from 2.9% reported in Turkey^75^ to 85.5% reported in Iran.^59^ In this meta‐analysis, the global pooled seroprevalence of *T. gondii* IgG antibody among neuropsychiatric patients was 38.27% (95% CI: 32.04%−44.9%). There was substantial heterogeneity with *I* ^2^ of 98.3% (Figure 2). Comparatively, lower global pooled seroprevalence of *T. gondii* IgG antibody was found among health controls (25.31%; 95% CI: 21.53−29.08). The seroprevalence of *T. gondii* IgG among healthy controls ranged from 0.7% reported in Lebanon 2020^44^ to 90% reported in Iran.^59^ Significant heterogeneity was also observed (*I* ^2^ value of 97.9%) (Figure 3).

### 3.4 Seroprevalence of acute *T. gondii* infection among neuropsychiatric patients and healthy controls

**Figure 4:**
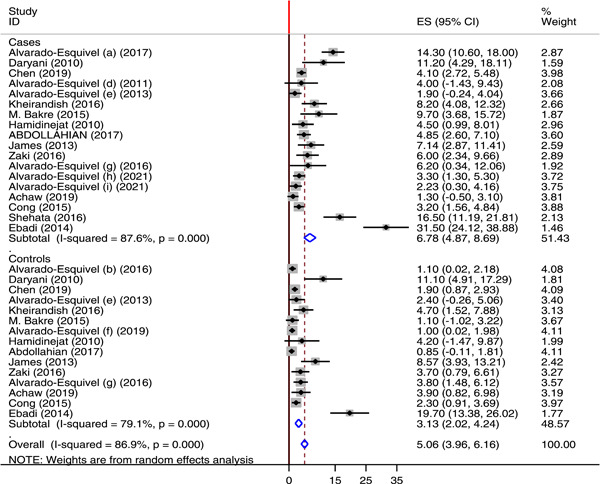
Forest plot showing the global seroprevalence of anti *Toxoplasma gondii* IgM among neuropsychiatric patients and healthy controls from 2010 to 2021.

Of the included 49 studies, 18 studies reported the prevalence of *T. gondii* IgM antibodies among neuropsychiatric patients and 15 studies reported the prevalence of IgM antibodies among healthy controls. The global pooled seroprevalence of *T. gondii* IgM antibody among neuropsychiatric patients was 6.78% with a range varying between 1.3% and 31.5%. On the other hand, the global pooled seroprevalence of *T. gondii* IgM antibody among healthy controls was 3.13% with a range varying between 0.85% and 19.7%. There was significantly high heterogeneity with *I* ^2^ value of 93.7% among the cases. However, the heterogeneity was relatively lower in the healthy controls (*I* ^2^ value of 79.1%) (Figure 4).

### 3.5 Subgroup analysis

**Figure 5:**
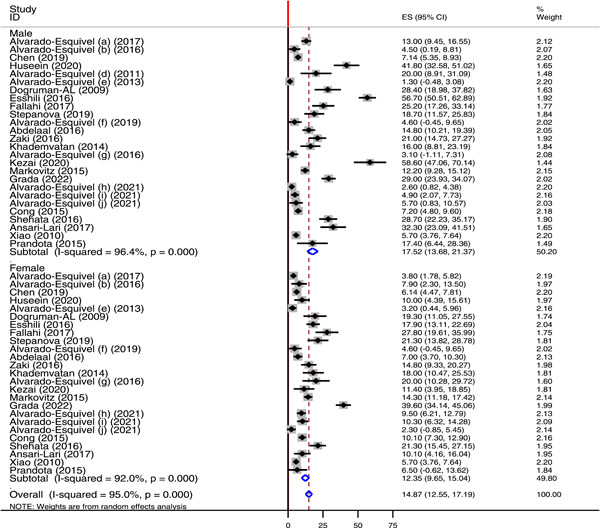
Forest plot showing the subgroup analysis *Toxoplasma gondii* IgG antibody seroprevalence by sex.

**Figure 6:**
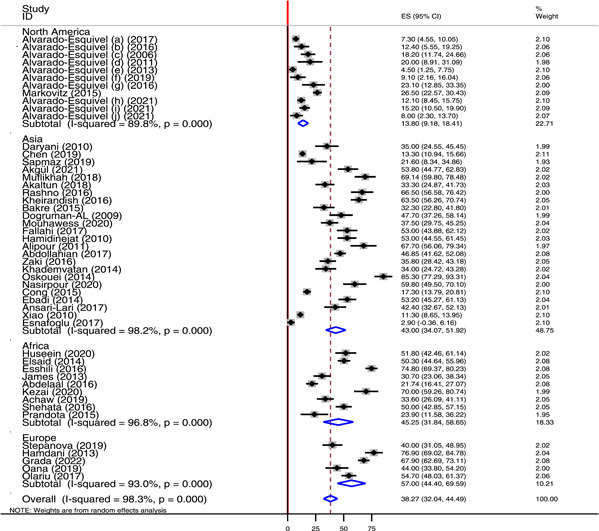
Forest plot showing the pooled prevalence of anti *Toxoplasma gondii* IgG in different region of the world.

**Figure 7:**
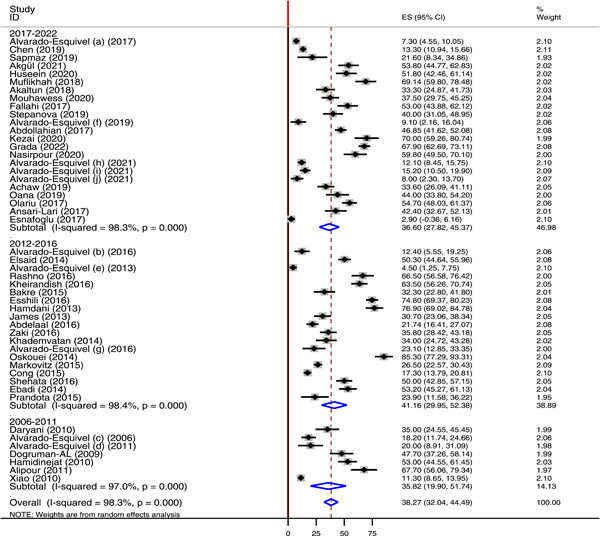
Forest plot showing the pooled seroprevalence of *Toxoplasma gondii* IgG antibody by publication year.

Subgroup analysis for sex, region, country, and publication year was done to investigate the source of heterogeneity across studies. With regard to sex of the study participants with neuropsychiatric disorders, 26 and 25 studies reported the prevalence of latent *T. gondii* infection among male and female patients, respectively. The prevalence of *T. gondii* IgG antibody was higher among males 17.52% (95% CI: 13.68−21.37) than in females 12.35% (95% CI: 9.65−15.04). In both cases, high heterogeneity was reported with *I* ^2^ of 96.4% and 92%, respectively (Figure 5). According to the continent, Europe contributed for the highest pooled seroprevalence of *T. gondii* IgG antibody (57%) followed by Africa (45.25%) and Asia (43%) (Figure 6). Another subgroup analysis was done for countries. According to this analysis, the highest pooled seroprevalence of *T. gondii* IgG antibody was found in Romania 56.1% followed by Iran (55.03%) and Egypt (36.93%). The least pooled prevalence was recorded in Mexico 11.85% (Table 2). Time based subgroup analysis showed the highest pooled prevalence of *T. gondii* IgG antibody in the year 2012−2016 (41.16%) than in 2006−2011 (36.6%) and 2017−2022 (35.82%) (Figure 7).

**Table 2:** Seroprevalence of anti *Toxoplasma gondii* IgG among different countries.

Country	Region	Number of studies	Pooled prevalence of IgG (%)	95% CI
Mexico	North America	10	11.85	8.54−15.16
Iran	Asia	12	55.03	46.88−63.19
China	Asia	3	13.76	10.7−16.82
Turkey	Asia	5	31.74	8.31−55.18
Egypt	Africa	4	36.93	19.75−54.11
Romania	Europe	3	56.1	42.97−69.23

### 3.6 Publication bias and sensitivity analysis

**Figure 8:**
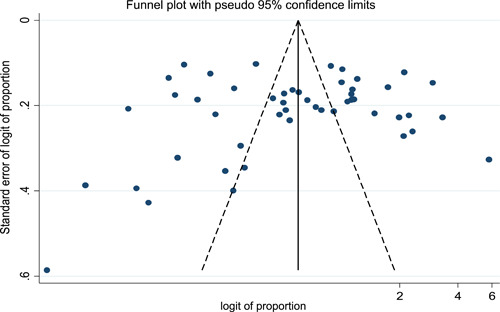
Funnel plot showing absence of publication bias.

In this study, the symmetry of the funnel plot indicated the absence of publication bias (Figure 8). Furthermore, the Egger's test statistics confirmed the absence of publication bias with *p*‐value of 0.765. According to sensitivity analysis, the pooled effect size when individual studies omitted lied within the 95% CI of the overall pooled effect size. This confirmed the absence of single study impact on the overall pooled seroprevalence of *T. gondii* infection.

## 4 DISCUSSION

The purpose of this systematic review and meta‐analysis was to determine the global seroprevalence of *T. gondii* infection among neuropsychiatric patients. To the best of our knowledge, our review represents the largest systematic review and meta‐analysis of the global pooled seroprevalence of *T. gondii* infection among neuropsychiatric patients including 21,093 study participants with 8592 neuropsychiatric patients and 12,501 healthy controls. People with neuropsychiatric disorders are highly affected by *T. gondii* infection proving the need for routine screening of neuropsychiatric patients for *T. gondii* infection. The present study revealed that the global pooled seroprevalence of chronic *T. gondii* infection was 38.27% (95% CI: 32.04−44.49) among neuropsychiatric patients and 25.31% (95% CI: 21.53−29.08) in apparently healthy controls with high heterogeneity (*I* ^2^ value of 98.3%). This finding is in line with the global pooled prevalence of *T. gondii* among HIV patients (35.8%; 95% CI: 30.8−40.7)^76^ and the national overall pooled prevalence of human *T. gondii* infection in Nigeria (32.92% [95% CI: 27.89−38.37]).^77^ *Toxoplasma gondii* induces a suite of behavioral and neurological changes in the infected individuals. There are pronounced behavioral changes in the individual infected with *T. gondii*. The parasite induced behavioral alteration include decreased cognitive function, prolonged reaction times, increased risk‐taking behavior, and suicidal tendencies in individuals with latent *T. gondii* infection.^32^, ^78^, ^79^ The reason for the complexity in human behavior is not yet understood, but it is commonly seen in *T. gondii* seropositive individuals.

The global seroprevalence of *T. gondii* IgG antibody among neuropsychiatric patients obtained in this study was higher than the pooled prevalence of *T. gondii* infection in cancer patients (30.8%),^80^ immunocompromised patients (35.9%),^81^ and Ethiopian general population (34.59%).^82^ On the other hand, the finding of this study was lower than the prevalence of *T. gondii* among the Iranian general population (39.3%).^83^ The observed variability might be due to differences in the study group, number of studies included in the meta‐analysis, and the diagnostic methods employed.

The high burden of *T. gondii* infection among patients with neurological and mental disorders can be associated with different mechanisms employed by this neurotropic parasite. Once passing the BBB through paracellular, transcellular or stowaway mechanism, *T. gondii* infection results in different spectrums of neuropsychiatric disorders and achieves this through alteration of the level of neurotransmitters. Majority of the neurobehavioral and neurological disorders associated with chronic toxoplasmosis are due to an elevation of dopamine in the brain and alteration in the level of GABA, serotonin, glutamate, nitric oxide, noradrenaline, and kynurenic acid.^18^ The neuropsychiatric disorders include; AD which is characterized by the abnormal configuration and excessive phosphorylation of amyloid deposition and neurofibrillary tangles in neurons. This is associated with *T. gondii* infection, due to the ability of the parasites to cause lower beta‐amyloid plaque deposition through inflammation induced beta‐amyloid phagocytosis and degradation.^84^ Unbalanced excitatory and inhibitory neurotransmission due to toxoplasmosis can induce seizure in *T. gondii* infected mice.^4^ Another neuropsychiatric disorder associated with *T. gondii* infection is bipolar disorder (manic depression), which encompasses a wide range of behavioral changes and involve reduction of corticosterone production due to latent *T. gondii* infection. Likewise, significantly higher prevalence of *T. gondii* infection was also reported among patients with schizophrenia. Toxoplasmosis patients are 2.73 times more likely to develop schizophrenia in their later life than healthy controls.^85^ The presence of large amount of bradyzoite in the olfactory bulb and production of large amount of anti *T. gondii* IgG is associated with the development of anosmia. A compressive review that assessed the association between *T. gondii* and neurologic disorders found that the increased production of nitric oxide (NO_2_), enhanced the production of inflammatory cytokines (interferon gamma, tumor necrosis factor alpha, interleukin 1 reactive oxygen and nitrogen species), neurotic biomolecules, and altered the dopamine balance leading to olfactory impairment, migraine, Asperger's syndrome, autism, and schizophrenia.^86^, ^87^

According to subgroup analysis, the pooled seroprevalence of *T. gondii* IgG antibody was higher among males 17.52% than in females 12.35%. This finding was in agreement with the finding of a meta‐analysis study that assessed toxoplasmosis in Iranian population.^83^ With regard to regional classification, a high burden of chronic *T. gondii* infection was reported in Europe (57%) followed by Africa (45.25%) and Asia (43%). The pooled prevalence of *T. gondii* IgG antibody in North America was 13.8%. The overall pooled seroprevalence of *T. gondii* IgG antibody among Iranian neuropsychiatric patient was 55.03% (95% CI: 46.88−63.19). This was higher than the prevalence of *T. gondii* among Iranian general population (39.3%),^83^ Iranian blood donors (34.4%),^88^ Iranian pregnant women (41%),^89^ and immunocompromised patients in Iran (50.01%).^90^ This study also showed 13.76% pooled seroprevalence of *T. gondii* IgG antibody among neuropsychiatric patients in China. This finding was higher than the prevalence of *T. gondii* among blood donors in China (6.26%).^91^ The variability in the seroprevalence of chronic toxoplasmosis across the different regions of the world and countries might be due to different reasons including geographical variation, difference in climatic condition, implementation of prevention and control strategies, personal and environmental hygienic conditions, and animal contact behaviors. In addition, difference in life style and habit of having close contact with cat or other felids can also contribute to the variability in the prevalence of *T. gondii* across the different regions and countries throughout the world.

The result of time‐based subgroup analysis demonstrated that the pooled prevalence of chronic *T. gondii* infection was higher in the year 2012−2016 (41.16%) compared to 2006−2011 (36.6%) and 2017−2022 (35.82%). The high prevalence in the year 2012−2016 might be due to climatic change, breaking of the prevention and control strategies, and environmental hygienic conditions.

The global pooled prevalence of acute *T. gondii* infection among neuropsychiatric patients and healthy controls was 6.78% (95% CI: 4.87−8.69) and 3.13% (95% CI: 2.02−4.24), respectively. This finding was higher than the global prevalence of acute toxoplasmosis among pregnant women (1.1%).^20^ According to the symmetry of the funnel plot and the Eggers test statistics (*p* value of 0.765) there was no publication bias in this systematic review and meta‐analysis.

The result of sensitivity analysis proved that there is no single study that affects the pooled effect size. The overall pooled prevalence of anti *T. gondii* IgG was calculated by omitting each study sequentially and the computed pooled prevalence was within the 95% CI of the overall pooled prevalence.

This study has few important limitations. First, the included studies were conducted only in 18 countries from Asia, Africa, North America, and Europe. There were no studies in Middle East, Latin America, and other corners of the world. Next, there was substantial heterogeneity observed between studies that may affect the interpretation of the results. Finally, studies conducted in a language other than English were excluded which might lead to lose of some studies to be included.

## 5 CONCLUSION

This systematic review and meta‐analysis showed that patients with neurological and psychiatric disorders are facing infection with the global endemic neurotropic parasite *T. gondii*. The global pooled prevalence of *T. gondii* IgG antibody in those patients was 38.27%. This urges clinicians to consider *T. gondii* infection in these patients, request appropriate routine testing to confirm the infection and provide appropriate treatment to those with the infection. Moreover, it is also an alarm to international, continental, and nation health bureaus and other stakeholders to develop targeted prevention and control strategies of *T. gondii* infection. This review also provides valuable information to policy makers and different stack holders. Moreover, the information could be used for future complimentary research.

## AUTHOR CONTRIBUTIONS

**Habtye Bisetegn**: Conceptualization; data curation; formal analysis; methodology; software; validation; visualization; writing—original draft. **Habtu Debash**: Methodology; resources; supervision; validation; visualization; writing—review and editing. **Hussen Ebrahim**: Formal analysis; investigation; methodology; project administration; validation; visualization; writing—review and editing. **Naunian Mahmood**: Formal analysis; methodology; software; supervision; visualization; writing—review and editing. **Alemu Gedefie**: Methodology; validation; visualization; writing—review and editing. **Mihret Tilahun**: Data curation; investigation; methodology; validation; visualization; writing—review and editing. **Ermiyas Alemayehu**: Conceptualization; investigation; methodology; validation; visualization; writing—review and editing. **Ousman Mohammed**: Conceptualization; formal analysis; investigation; methodology; software; writing—review and editing. **Daniel Getacher Feleke**: Formal analysis; investigation; methodology; validation; visualization; writing—review and editing.

## CONFLICT OF INTEREST STATEMENT

The authors declare no conflict of interest.

## TRANSPARENCY STATEMENT

The lead author Habtye Bisetegn affirms that this manuscript is an honest, accurate, and transparent account of the study being reported; that no important aspects of the study have been omitted; and that any discrepancies from the study as planned (and, if relevant, registered) have been explained.

## Data Availability

All data required for this research are available within the manuscript. If additional data are needed, it can be obtained from the corresponding authors upon request.
